# A CT-based interpretable machine learning model for preoperative prediction of pancreatic neuroendocrine tumor aggressiveness

**DOI:** 10.3389/fonc.2025.1665601

**Published:** 2025-12-11

**Authors:** Rong Kong, Shunzu Lu, Yugui Huang, Siyu Tan, Chunxia Zhu, Guowei Chen, Mingrui Yang, Ying Liu, Qixin Wu, Peng Peng

**Affiliations:** 1 Department of Radiology, the First Affiliated Hospital of Guangxi Medical University, Nanning, Guangxi, China; 2 Department of Geriatric Gastroenterology, the First Affiliated Hospital of Guangxi Medical University, Nanning, Guangxi, China; 3 Department of Radiology, Chongzuo People’s Hospital, Chongzuo, Guangxi, China

**Keywords:** pancreatic neuroendocrine tumor, aggressiveness, computed tomography, machine learning, interpretable model, shap, preoperative prediction

## Abstract

**Objectives:**

This study aimed to develop and validate an interpretable machine learning (ML) model based on structured preoperative CT features for non-invasive prediction of pancreatic neuroendocrine Tumors (PNETs) aggressiveness.

**Methods:**

This retrospective study included 112 patients with PNETs who underwent contrast-enhanced abdominal CT. Patients were randomly assigned to training and validation cohorts. Clinical data and CT features were analysed using the Least Absolute Shrinkage and Selection Operator method and multivariate logistic regression to identify independent risk factors. Multiple ML models were evaluated to determine the optimal classifier. Model performance was assessed using receiver operating characteristic and calibration curves, and decision curve analysis. Shapley Additive Explanations (SHAP) quantified feature importance for interpretable risk prediction.

**Results:**

A total of 112 patients were evaluated, including 80(mean age± standard deviation, 47 ± 13 years; 36 males)) in the training set and 32 (48 ± 15 years; 12 males) in the validation set. Tumour shape, necrotic changes, arterial relative enhancement ratio, and enhancement pattern independently predicted PNETs aggressiveness. The logistic regression model demonstrated excellent discrimination, achieving an area under the curve of 0.952 (95% CI: 0.952 (0.909–0.994) in the training cohort and 0.972 (95% CI 0.927–1.000) in the validation cohort. SHAP summary and force plots facilitated global and local model interpretation.

**Conclusion:**

The Interpretable ML model based on CT features could serve as a preoperative, noninvasive, and precise evaluation tool to differentiate aggressive and non-aggressive PNETs, facilitating personalized clinical management and potentially improving patient outcomes.

## 1 Introduction

Pancreatic neuroendocrine Tumors (PNETs), which originate from neuroendocrine cells, comprising approximately 5% of pancreatic tumors and exhibiting considerable clinical heterogeneity (1, 2). Although they are uncommon, the incidence has shown a yearly increasing trend over the past three decades (3, 4). Previously considered indolent, recent studies reveal their complexity, with some subtypes being highly aggressive (5). Studies have demonstrated that while the median survival for localized NETs exceeds 30 years, those with peritoneal metastases face a drastically reduced median survival of only about 1 year (6, 7). Given their potential for aggressive behavior, PNETs require careful clinical evaluation and management.

Surgery remains the primary and only curative treatment (8); however, no consensus exists on the scope of surgical intervention, partly due to challenges in assessing tumour aggressiveness preoperatively. Treatment strategies and prognoses vary with tumour aggressiveness. For non-aggressive PNETs, local resection lowers surgical risks without affecting prognosis, while aggressive PNETs require radical resection, lymph node dissection, and adjuvant therapy to minimise recurrence. Thus, precise preoperative assessment of tumour invasiveness is critical for optimising clinical decisions and tailoring surgical plans.

Currently, the TNM staging system and World Health Organisation (WHO) histopathological classification assess invasiveness; however, these systems rely on postoperative data, limiting preoperative utility (9, 10). Endoscopic ultrasound-guided fine-needle aspiration biopsy (EUS-FNAB), commonly used in PNETs, may help risk-stratify patients by determining tumour histological grade. However, it is invasive, carries potential risks, and has limited accuracy (11), making reliable histology obtainable only postoperatively. Thus, a non-invasive and repeatable method for preoperative PNETs aggressiveness prediction is urgently needed, yet no widely accepted solution exists.

Recent advancements in artificial intelligence, particularly machine learning (ML), enable high-precision predictions by analysing data and identifying patterns through powerful algorithms. These technologies are transforming biomedical research, personalised medicine, and computer-aided diagnosis (12). Compared to traditional methods, ML-based feature selection models offer superior predictive capabilities across diseases. By analysing complex imaging and clinical data, these models assist in predicting PNETs invasiveness and optimising clinical management. Although ML algorithms are promising, their “black box” nature, particularly the lack of interpretability in risk prediction models, limits clinical application (13, 14).

The Shapley Additive Explanation (SHAP) method, a practical decision-making explaining tool, quantifies features-outcome relationships, improving model interpretability (15). It generates personalised risk predictions and visualises each feature’s contribution, integrating biological and clinical models. This approach enhances clinicians’ understanding of predictive models while supporting personalised medicine. To our knowledge, the noninvasive aggressiveness prediction in PNETs using clinical and CT features based on interpretable machine learning has not been well established in the literature.

This study aimed to develop and validate a non-invasive ML model using CT-based quantitative and qualitative features alongside clinical factors to predict PNETs invasiveness preoperatively. Additionally, the SHAP method was employed to visualise and explain features-clinical outcome relationships, improving clinician confidence in predictions and enabling early interventions. To our knowledge, no previous study has combined CT features with ML and utilised SHAP to predict PNETs aggressiveness.

## 2 Materials and methods

This retrospective study was approved from the institutional ethics committees and waived the requirement for written informed consent from the patients.

### 2.1 Patients

Patients with pathologically confirmed PNETs treated at the First Affiliated Hospital of Guangxi Medical University between May 2015 and May 2024 were included. Exclusion criteria were: (1) local or systemic therapy before imaging, (2) Absence of preoperative CT or an interval exceeding 4 weeks between CT and surgery, (3) Missing pathological grade information, (4) Poor image quality unsuitable for analysis.

**Figure 1:**
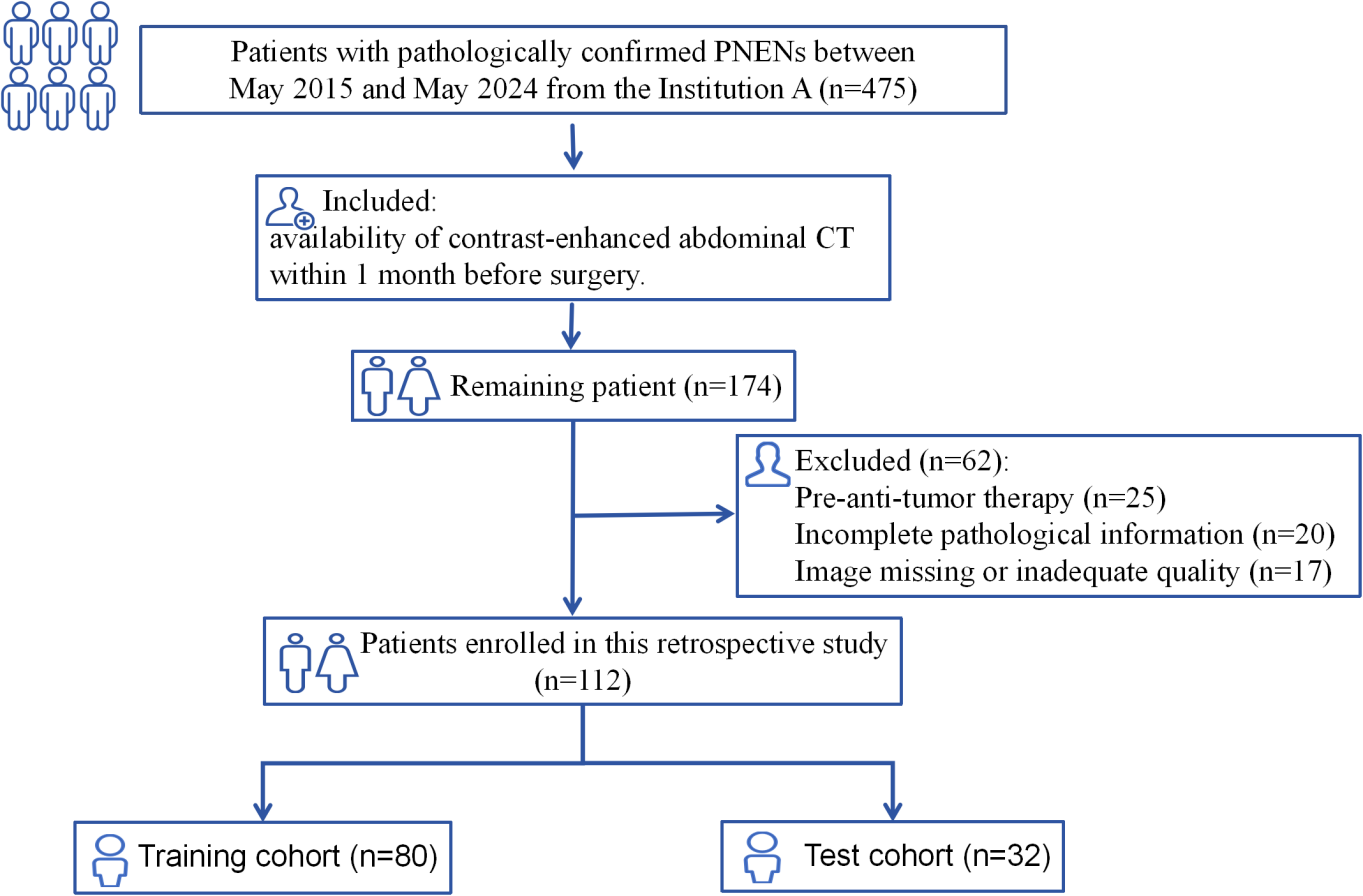
Flowchart illustrating the patient selection process in this study.

The study recruitment process is illustrated in **Figure 1**. Patients were randomly assigned to training and test cohorts. Baseline clinical data, including sex, age, symptoms (present/absent), alpha-fetoprotein, carcinoembryonic antigen, carbohydrate antigens (CA125, CA153, CA199), and haematocrit levels, were collected from medical records.

### 2.2 Definition of PNETs aggressiveness

PNETs aggressiveness was determined based on postoperative histopathological and clinical outcomes. Tumours were classified as aggressive if any of the following endpoints were present: G3 tumour grade, distant metastases, metastatic lymph nodes, microvascular invasion, and/or disease recurrence (16). PNETs were graded per WHO 2017 criteria: G1: Ki-67 ≤2%, G2: Ki-67 = 3–20%, G3: Ki-67 >20% (17). Metastatic lymph nodes status(N+) was assigned only for patients who underwent lymph node dissection with histologically confirmed nodal metastasis. Disease recurrence was evaluated during follow-up through outpatient visits or telephone interviews and was defined as radiologically or pathologically confirmed local recurrence or distant relapse after curative resection. Both the site and number of recurrences lesions were recorded.

### 2.3 CT protocols

Patients fasted from solid food for 4–6 h. Owing to the extended data collection period, multiple CT scanners were used: 64-channel CT (LightSpeed VCT, GE Healthcare), 256-channel CT (Revolution, GE Healthcare), and a dual-source CT (SOMATON Definition Flash, Siemens Healthcare). Scan parameters were: tube voltage, 120 kV; tube current: 250–350 mA; matrix, 512 × 512; and slice thickness, 2.0–2.5mm. For contrast-enhanced imaging, a non-ionic contrast medium (iohexol, iodine content, 350 mg/mL) was administered intravenously at 3 mL/s via an automatic power injector. Pre-contrast scans were followed by contrast-enhanced scans at 35–45 s (arterial phase), 70 s (portal venous phase), and 180 s (equilibrium phase) post-contrast injection.

### 2.4 CT feature analysis

#### 2.4.1 Qualitative features

CT images were retrieved from the picture archiving and communication system (PACS) and reviewed by two radiologists (6 and 10 years of experience in abdominal diagnosis), both of whom were aware of the presence of PNETs but were blinded to clinical and pathological data. Discrepancies in assessment were resolved by consensus following consultation with a third radiologist with 25 years of clinical experience in pancreatic imaging.

The following qualitative tumour features were assessed: location, size, number, shape, components, margin, and presence of necrosis. Other features are calcification, homogeneity, biliopancreatic duct dilatation, pancreatic parenchymal atrophy, uniformity of enhancement, and enhancement patterns. When multiple lesions were present, the tumour with the largest diameter was selected for analysis.

For tumour margins, a smooth, visible edge was considered well-defined, while spiculation or infiltration along more than 25% of the tumour perimeter was considered ill-defined (18). Uniformity of enhancement, assessed on portal phase images, was categorised as homogeneous or heterogeneous (19). The main pancreatic duct was classified as dilated when its diameter was ≥3 mm, and the common bile duct was considered dilated when its diameter was ≥10 mm (20). On contrast-enhanced images, tumours were categorised according to enhancement patterns as either hyper-attenuating (hyper-group) or iso/hypo-attenuating alone (iso/hypo-group) (21).

#### 2.4.2 Quantitative assessment

The CT values (in Hounsfield unit, HU) values were measured using manually drawn circular/ovoid regions of interest (ROI), carefully avoiding calcifications, haemorrhage, cystic or necrotic components, vessels, and artefacts. ROIs were copied and pasted across phases of the same area with necessary adjustments. Serum haematocrit levels were obtained within 48 h of CT. Intra-observer agreement was ensured by repeating measurements, with the mean used for analysis.

Quantitative assessment was performed as follows: (a) Absolute enhancement = HU (arterial/portal phase) – HU (unenhanced tumour) (22); (b) Relative enhancement ratio: Arterial/portal enhancement divided by HU (unenhanced phase) (22); (c) Enhancement ratio: HU (arterial/portal tumour) divided by HU (adjacent parenchyma) on the same phase (20); (d) Extracellular volume fraction (ECV) was calculated using the formula: ECV (%) = (100 - hematocrit) × (ΔHU _tumour_/ΔHU _aorta_), where ΔHU _tumour_ and ΔHU _aorta_ are the HU values on equilibrium-phase images minus those on pre-contrast images for the tumour and abdominal aorta, respectively (23).

### 2.5 Feature selection and model construction

Patients were randomly divided (7:3 ratio) into training and validation cohorts. Least Absolute Shrinkage and Selection Operator (LASSO) regression with 10-fold cross-validation was used to optimise the variable selection and model complexity. The optimal λ value was determined to minimise cross-validation error based on non-zero coefficients, addressing multicollinearity via L1 regularisation. Subsequently, a multivariate logistic regression was applied once within the training cohort to identify independent predictors from the LASSO-selected variables. This step was used exclusively for feature refinement and did not constitute model training.

After feature selection, seven commonly used ML classifiers—Logistic Regression, Extreme Gradient Boosting (XGBoost), AdaBoost, K-Nearest Neighbors (KNN), Neural Network, Support Vector Machine (SVM), and CatBoost—were trained using only the selected predictors. All models were trained using repeated 10-fold cross-validation (10-fold CV with 5 repeats) within the training cohort to enhance model stability and reduce variance. Model performance was evaluated in both the training and validation cohorts.

Receiver-operating characteristic (ROC) curves and the area under the curve (AUC) were used to assess predictive accuracy for PNETsinvasiveness. Pairwise AUC comparisons among the ML models were conducted using DeLong’s test with Bonferroni correction. Additional evaluation metrics included accuracy, F1-score, sensitivity, and specificity. Calibration curves assessed predict reliability, while Decision Curve Analysis (DCA) evaluated the clinical applicability of the models (24). The optimal model was selected based on a comprehensive evaluation of discrimination, calibration, cross-cohort stability, and clinical net benefit.

### 2.6 Model interpretation and visualisation

To improve model interpretability, the SHAP method was applied. SHAP allows for both cohort- and patient-level interpretations by aggregating and averaging SHAP values to assess each feature’s impact (15). The SHAP feature importance plot ranks the features based on their overall contribution to the model. The SHAP bee swarm plot displays the range and distribution of each feature’s contribution, associating feature values with their corresponding impacts on the model. SHAP dependence plots illustrate how changes in feature values affect model predictions, while SHAP force plots provide an intuitive visualisation of how individual features influence a single prediction.

### 2.7 Statistical analysis

All statistical analyses were performed using SPSS (version 26.0, IBM) and R software (version 4.4.2). Continuous variables were expressed as mean (SD) and compared using an unpaired, two-tailed t-test (continuous variables with a normal distribution) or the Mann-Whitney test (continuous variables without a normal distribution). Categorical variables were compared using the χ^2^ test or Fisher’s exact test. In all analyses, *p* < 0.05 was considered statistically significant.

## 3 Results

### 3.1 Clinical and baseline characteristics

The study included 112 patients, randomly divided into training (n = 80; mean age, 47 ± 13 years; 36 males) and test (n = 32; mean age, 48 ± 15 years; 12 males) sets. The proportion of aggressive tumours was 41.25% (33/80) and 40.62% (13/32) in the training and test sets, respectively. In the training set, the aggressive group included patients with G3 grading (n = 11), lymph node metastasis (n = 17), microvascular invasion (n = 5), preoperative distant metastasis or postoperative recurrence (n = 24). Liver metastasis was the most common site of recurrence (n = 22), followed by local recurrence (n = 2). Similarly, in the test set, the aggressive group included patients with G3 grading (n = 4), lymph node metastasis (n = 5), microvascular invasion (n = 3), and preoperative distant metastasis or postoperative recurrence (n = 8). Liver involvement was again the predominant site (n = 7), followed by local recurrence (n = 1). The sum of individual components exceeded the number of aggressive cases in both cohorts because some patients had multiple aggressiveness endpoints. No significant differences were observed between the training and test cohorts (**Table 1**).

**Table 1:** Baseline characteristics of the training and validation cohorts.

Variables	Levels	Total	Training set	Test set	*P-*value
		(n = 112)	(n =80)	(n = 32)	
Gender, n (%)	Female	64 (57.14)	44 (55.00)	20 (62.50)	0.469
	Male	48 (42.86)	36 (45.00)	12 (37.50)	
Age, years		47.22 ± 13.16	47.09 ± 12.65	47.56 ± 14.56	0.864
Syndrome, n (%)	NO	80 (71.43)	59 (73.75)	21 (65.62)	0.390
	YES	32 (28.57)	21 (26.25)	11 (34.38)	
^a^AFP		2.60 (1.98, 3.80)	2.71(2.00, 3.86)	2.54 (1.88, 3.29)	0.680
^b^CEA		1.76 (1.16, 3.01)	1.73(1.15, 2.80)	1.98 (1.26, 3.38)	0.735
^c^CA 125		13.05 (8.30, 26.93)	13.25 (9.90, 30.12)	12.40 (7.70, 22.18)	0.350
^d^CA 153		11.65 (7.88, 14.40)	11.10(8.02, 14.30)	12.15 (7.88, 16.05)	0.357
^e^CA 199		7.89 (2.85, 18.00)	7.22(2.75, 15.98)	8.24 (3.62, 25.01)	0.440
Tumour location, n (%)	Head/neck	50 (44.64)	36 (45.00)	14 (43.75)	0.904
	Body/tail	62 (55.36)	44 (55.00)	18 (56.25)	
Tumour size		3.80 (2.20, 5.67)	3.80 (2.45, 5.60)	3.65 (1.87, 5.70)	0.750
Tumour shape, n (%)	Regular	80 (71.43)	57 (71.25)	23 (71.88)	0.947
	Irregular	32 (28.57)	23 (28.75)	9 (28.12)	
Tumour components, n (%)	Solid	105 (93.75)	74 (92.50)	31 (96.88)	0.666
	Solid-cyst	7 (6.25)	6 (7.50)	1 (3.12)	
Tumour Margin, n (%)	Well-defined	65 (58.04)	47 (58.75)	18 (56.25)	0.809
	Ill-defined	47 (41.96)	33 (41.25)	14 (43.75)	
Tumour number, n (%)	Solitary	108 (96.43)	76 (95.00)	32 (100.00)	0.469
	Multiple	4 (3.57)	4 (5.00)	0 (0.00)	
Necrosis, n (%)	Absence	49 (43.75)	35 (43.75)	14 (43.75)	1.000
	Presence	63 (56.25)	45 (56.25)	18 (56.25)	
Calcification, n (%)	Absence	87 (77.68)	59 (73.75)	28 (87.50)	0.114
	Presence	25 (22.32)	21 (26.25)	4 (12.50)	
Homogeneity, n (%)	Homo-	55 (49.11)	38 (47.50)	17 (53.12)	0.591
	Hetero-	57 (50.89)	42 (52.50)	15 (46.88)	
Uniformity of enhancement, n (%)	Homo-	50 (44.64)	37 (46.25)	13 (40.62)	0.589
	Hetero-	62 (55.36)	43 (53.75)	19 (59.38)	
Pancreatic duct expansion, n (%)	NO	79 (70.54)	57 (71.25)	22 (68.75)	0.793
	YES	33 (29.46)	23 (28.75)	10 (31.25)	
Pancreatic atrophy, n (%)	NO	98 (87.50)	72 (90.00)	26 (81.25)	0.343
	YES	14 (12.50)	8 (10.00)	6 (18.75)	
Enhancement pattern, n(%)	Hyper	61 (54.46)	44 (55.00)	17 (53.12)	0.857
	Iso/hypo	51 (45.54)	36 (45.00)	15 (46.88)	
Arterial absolute enhancement		80.5(47.75, 119.25)	77.5(47.75, 130.0)	82.0(46.75,105.0)	0.735
Portal absolute enhancement		70.0(54.75, 109.25)	71.5(56.00, 107.5)	67.5(46.25,119.0)	0.706
Arterial relative enhancement ratio		1.80 (1.03, 2.71)	1.83 (1.09, 3.01)	1.72 (0.98, 2.27)	0.573
Portal relative enhancement ratio		1.64 (1.25, 2.56)	1.68 (1.36, 2.56)	1.49 (1.11, 2.55)	0.406
Arterial enhancement ratio		1.15 (0.87, 1.41)	1.17 (0.90, 1.44)	1.10 (0.85, 1.30)	0.307
Portal enhancement ratio		1.13 (0.95, 1.32)	1.14 (0.98, 1.33)	1.10 (0.92, 1.26)	0.534
^f^ECV		48.37(41.64, 55.82)	48.10(41.64,55.84)	50.34(42.27,55.82)	0.671
Result, n (%)	No	66 (58.93)	47 (58.75)	19 (59.38)	0.952
	Yes	46 (41.07)	33 (41.25)	13 (40.62)	
Grade	G1/G2	97(86.61)	69(86.25)	28(87.5)	1.000
	G3	15(13.39)	11(13.75)	4(12.5)	
Microvascular invasion	NO	104(92.86)	75(93.75)	29(90.62)	0.863
	Yes	17(15.18)	11(13.75)	5(9.38)	
Recurrence					
Local recurrence		3(2.68)	2(2.5)	1(3.12)	1.000
Liver metastasis		29(25.89)	22(27.5)	7(21.88)	0.539
Lymph node metastasis	No	90(80.36)	63(78.75)	27(84.38)	0.498
	Yes	22(19.64)	17(21.25)	5(15.62)	

^a^AFP, alpha-fetoprotein; ^b^CEA, carcinoembryonic antigen, ^c^CA125 carbohydrate antigen 125, ^d^CA153 carbohydrate antigen 153, ^e^CA199 carbohydrate antigen 199, ^f^ECV extracellular volume fraction.

### 3.2 Feature extraction and selection

**Figure 2:**
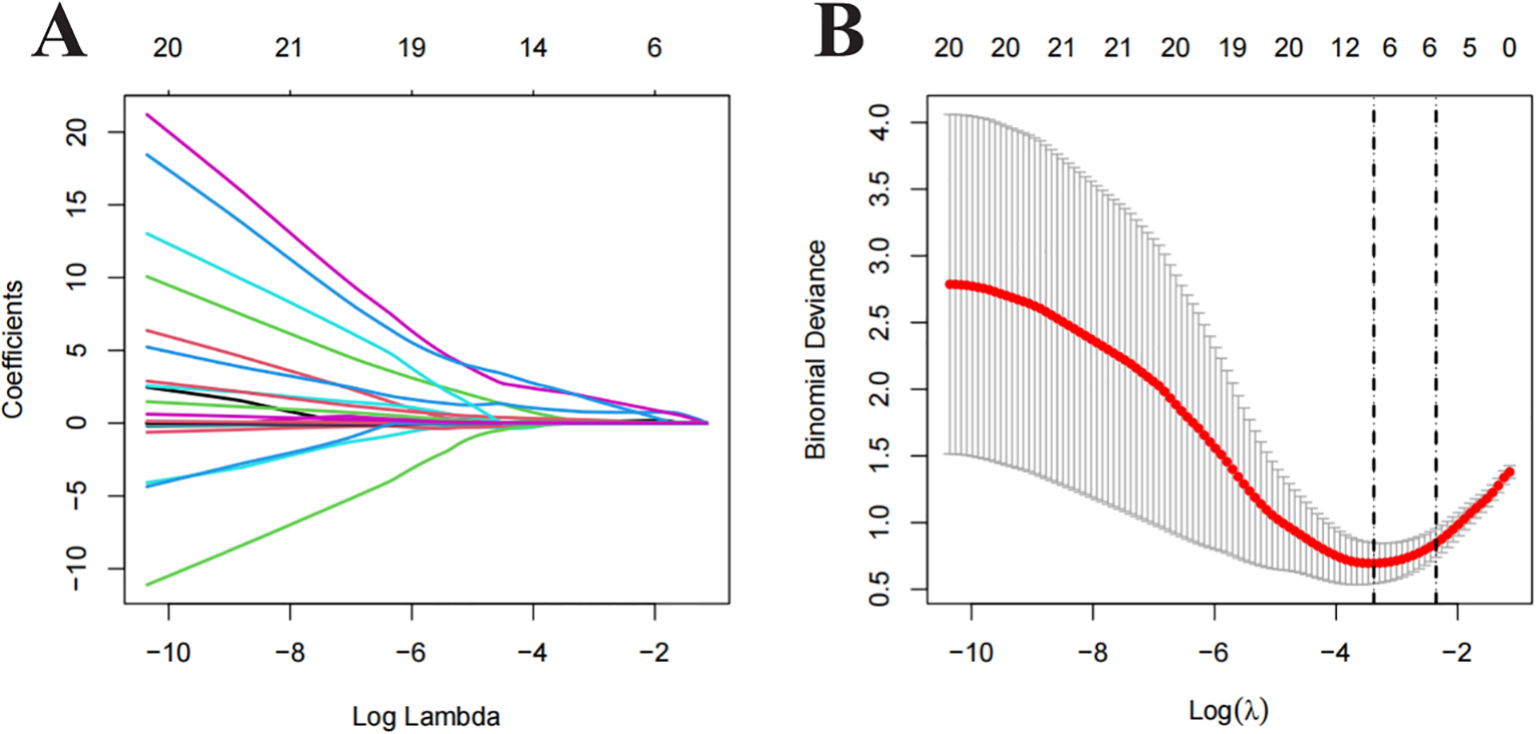
Least Absolute Shrinkage and Selection Operator (LASSO) regression analysis for feature selection. **(A)** Distribution of LASSO coefficients for different λ values. **(B)** Optimal λ values selected based on 10-fold cross-validation and minimum mean squared error (MSE), represented by vertical dashed lines.

**Figure 3:**
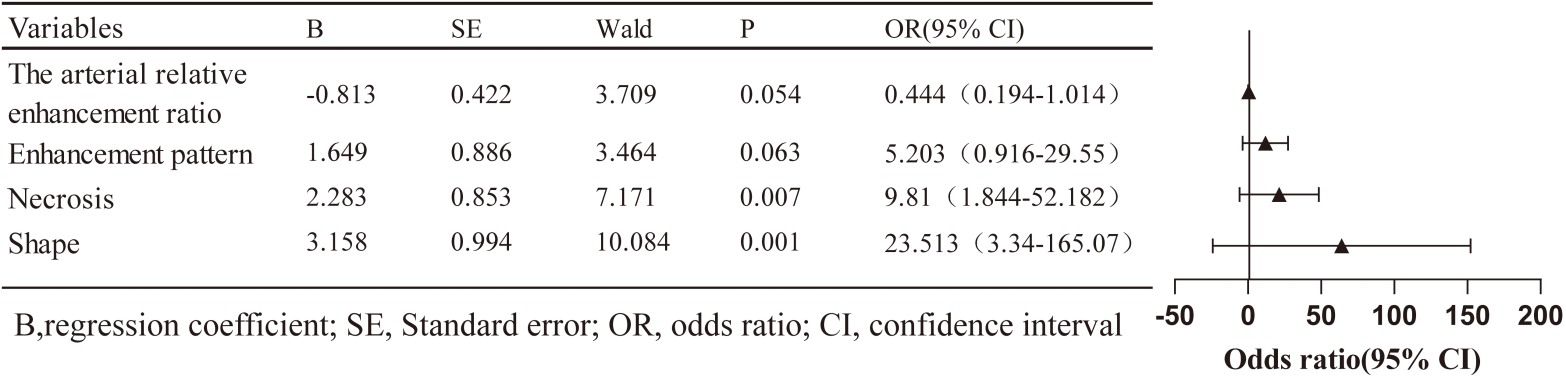
Forest plot showing the results of multivariate logistic regression analysis.

All clinical and imaging features were analysed using LASSO regression (**Figure 2**). The model identified 12 predictive factors using the minimum λ value of 0.034: tumour location, shape, margin, necrosis, pancreatic duct expansion, pancreatic atrophy, enhancement pattern, size, CA153, CA199, arterial absolute enhancement, and arterial relative enhancement ratio. To control for confounding variables, multivariable logistic regression identified tumour shape (OR = 23.513, 95% CI: 3.340–165.070), necrosis (OR = 9.810, 95% CI: 1.844–52.182), enhancement pattern (OR = 5.203, 95% CI: 0.916–29.550), and arterial relative enhancement ratio (OR = 0.444, 95% CI: 0.194–1.014) as independent predictors of invasiveness (**Figure 3**). These factors were incorporated into ML models.

### 3.3 Development and validation of ML models

Model performance metrics, including AUC, accuracy, F1 score, sensitivity, and specificity, are summarised in **Table 2**. In the training set, the KNN classifier yielded the highest AUC (0.994; 95% CI, 0.983–1.000), followed by CatBoost (0.969; 95% CI, 0.938–1.000) and logistic regression (0.952; 95% CI, 0.909–0.994), with SVM, XGBoost, Neural Network, and Adaboost slightly lower. In the validation set, the logistic regression model achieved the highest AUC (0.972, 95% CI: 0.927–1.000), followed by SVM (0.947, 95% CI: 0.879–1.000), CatBoost (0.941, 95% CI: 0.865–1.000), Neural Network (0.907, 95% CI: 0.792–1.000), Adaboost (0.919, 95% CI: 0.828–1.000), XGBoost (0.891, 95% CI: 0.782–1.000), and KNN (0.848, 95% CI: 0.712–0.984). Regarding accuracy, the logistic regression model, SVM, Neural Network, XGBoost, KNN, Adaboost, and CatBoost achieved 90.6%, 87.5%, 90.6%, 78.1%, 81.2%, 87.5%, and 87.5% accuracy, respectively. Logistic regression also achieved the highest F1 score (0.897), balanced sensitivity (100%) and specificity (0.842) in the validation cohort.

**Table 2:** Diagnostic performance of machine learning classifiers in predicting pancreatic neuroendocrine neoplasm aggressiveness.

Cohort	Model	^a^AUC (95% CI)	Accuracy	Sensitivity	Specificity	Precision	F1-score
Training set	Logistic	0.952(0.909–0.994)	0.900	0.879	0.915	0.879	0.879
	^b^SVM	0.945(0.898–0.993)	0.912	0.909	0.915	0.882	0.896
	Neural Network	0.928(0.874–0.983)	0.863	0.879	0.851	0.806	0.841
	^c^XGboost	0.947(0.902–0.992)	0.900	1.000	0.830	0.805	0.892
	^d^KNN	0.994(0.983–1.000)	0.975	0.939	1.000	1.000	0.969
	Adaboost	0.942(0.894–0.989)	0.875	0.879	0.872	0.829	0.853
	CatBoost	0.969(0.938–1.000)	0.925	0.970	0.894	0.865	0.914
Validation set	Logistic	0.972(0.927–1.000)	0.906	1.000	0.842	0.812	0.897
	^b^SVM	0.947(0.879–1.000)	0.875	0.923	0.842	0.800	0.857
	Neural Network	0.907(0.792–1.000)	0.906	0.769	1.000	1.000	0.87
	^c^XGboost	0.891(0.782–1.000)	0.781	0.923	0.684	0.667	0.774
	^d^KNN	0.848(0.712–0.984)	0.812	0.923	0.737	0.706	0.800
	Adaboost	0.919(0.828–1.000)	0.875	0.769	0.947	0.909	0.833
	CatBoost	0.941(0.865–1.000)	0.875	0.846	0.895	0.846	0.846

^a^AUC, area under the curve; ^b^SVM, Support Vector Machine; ^c^XGBoost, extreme Gradient Boosting; ^d^KNN, K-Nearest Neighbors.

**Figure 4:**
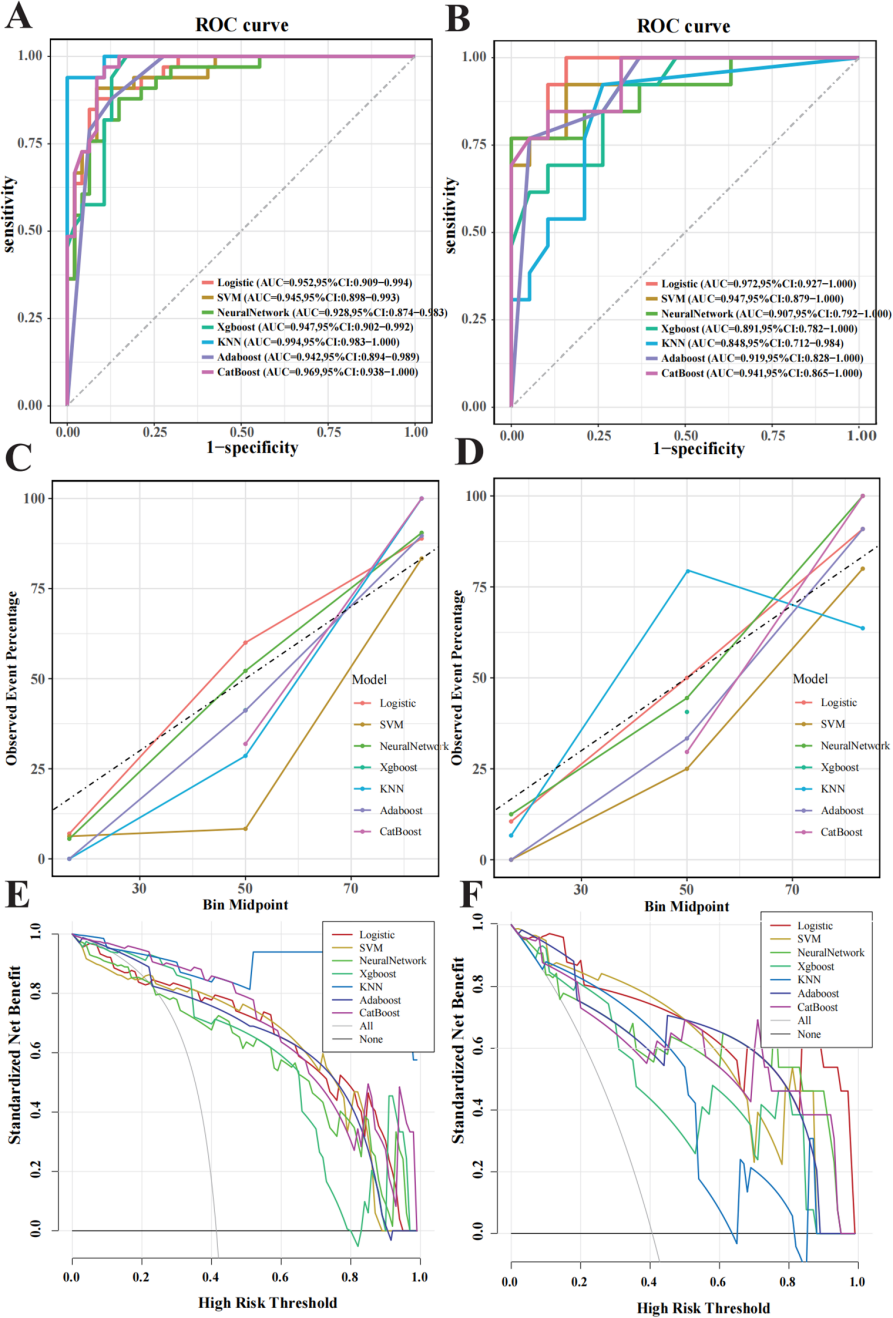
Performance evaluation of the machine learning model in predicting pancreatic neuroendocrine neoplasm invasiveness. **(A)** Receiver operating characteristic (ROC) curves for the training set. **(B)** ROC for the validation set. **(C)** Calibration curves for the training set, with the x-axis representing average predicted probability and the y-axis representing actual event probability. **(D)** Calibration curve for the validation set. **(E)** Decision Curve Analysis (DCA) curves for the training set. **(F)** DCA curve for the validation set.

ROC curves were generated for both the training and validation sets to evaluate confirmed the model’s performance (**Figures 4A, B**). In the training set, KNN, CatBoost, and logistic regression models performed the best, while the logistic regression model showed the highest performance in the testing set, with an AUC of 0.972 (95% CI: 0.927–1.000). Calibration curves (**Figures 4C, D**) demonstrated good agreement between predicted and observed tumour invasiveness in both sets, indicating accurate predictions. DCA demonstrated a high net benefit across threshold probabilities (**Figures 4E, F**). Pairwise comparisons of AUC values among the seven classifiers showed no statistically significant differences (all p > 0.05). Nonetheless, logistic regression was selected as the optimal model based on its multidimensional strengths, including superior discrimination, accurate calibration, cross-cohort stability, and consistently high clinical utility. Together, these findings support logistic regression as the most robust and clinically applicable model for predicting PNETs invasiveness.

### 3.4 Exploration of model interpretability

**Figure 5:**
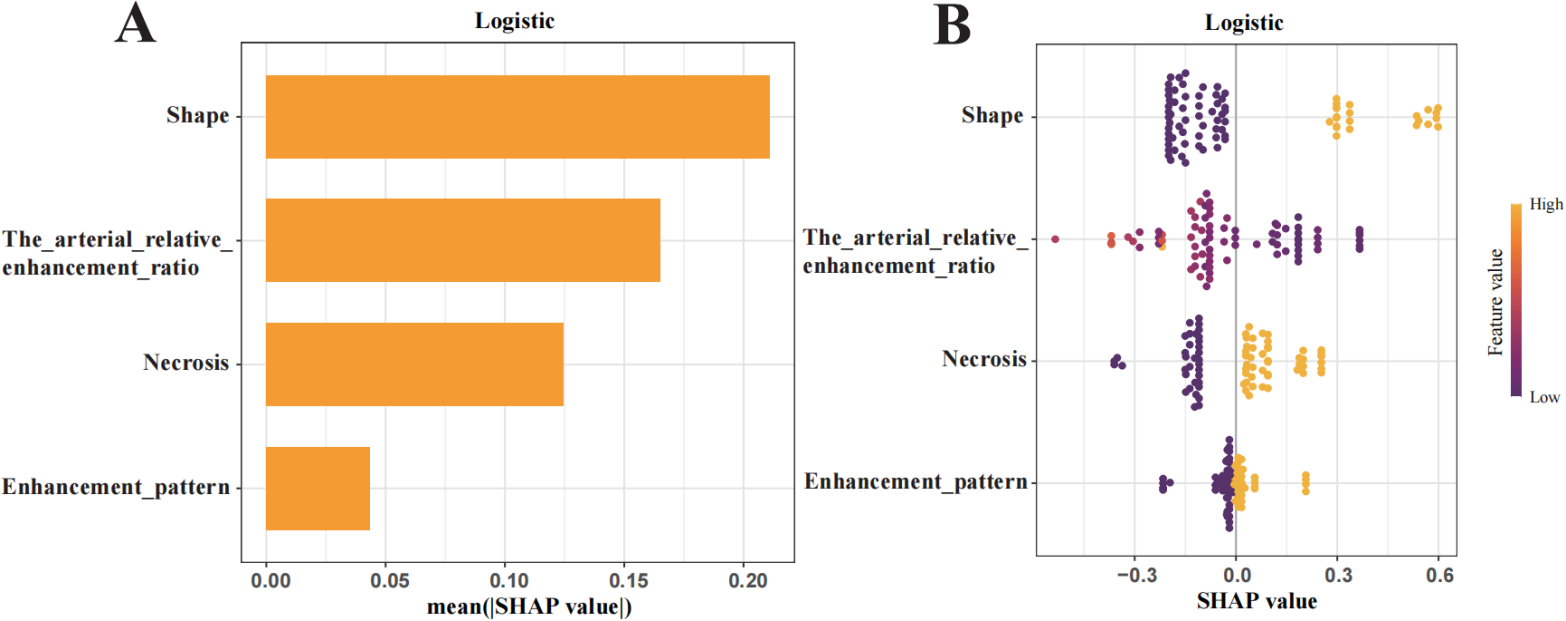
Shapley Additive Explanations (SHAP) summary plot illustrating feature importance. **(A)** Ranking of feature importance in the final predictive model. **(B)** SHAP values of each feature in the final model, where each line represents a feature, and the abscissa is the SHAP value. The different colors (yellow and purple) represent different levels of effect on the output of the model.

**Figure 6:**
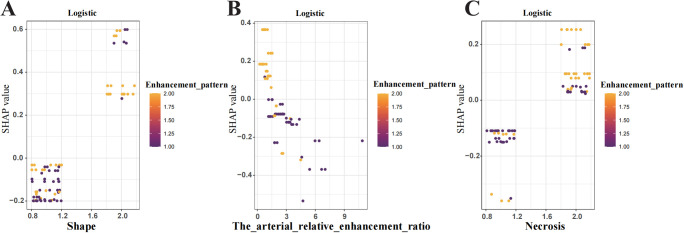
Shapley Additive Explanations (SHAP) dependence plot shows the influence of individual features on model predictions. **(A)** Tumour shape **(B)** Arterial relative enhancement ratio change **(C)** Tumour necrosis. The y-axis represents SHAP values, while the x-axis displays specific feature values. Each point represents the SHAP value for a patient, with the colour gradient from yellow to purple indicating high to low feature values. A SHAP value >0 indicates an increased risk of tumour aggressiveness.

To enhance interpretability, SHAP values were used to assess features importance. The top-ranked predictors in the logistic regression model were tumour shape, arterial relative enhancement ratio, necrosis, and enhancement pattern (**Figure 5A**). The SHAP bee swarm plot (**Figure 5B**) illustrates the contribution of each feature, with purple indicating a negative effect and yellow indicating a positive effect on the predicted probability. Each point represents a patient’s SHAP value, with the density of the points reflecting the distribution of the same SHAP value. The points are coloured according to the feature values, ranging from low (purple) to high (yellow). For instance, patients with irregular shapes or lower arterial relative enhancement ratios are more likely to have invasive tumours. The SHAP dependency plot (**Figure 6**) demonstrates that lower arterial relative enhancement ratios correlated with increased tumour invasiveness. Irregular tumour shape and necrosis also correlated with increased SHAP values.

**Figure 7:**
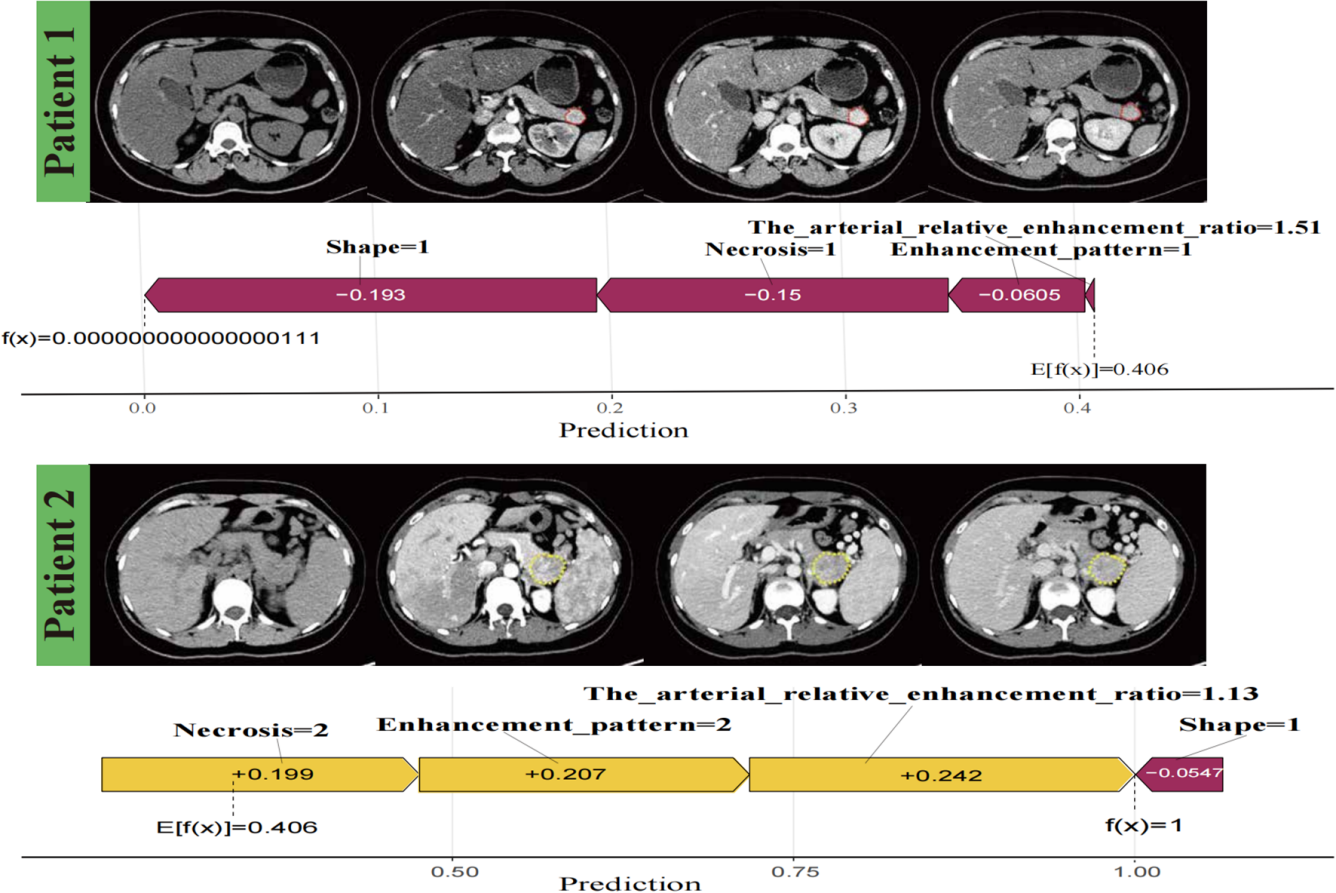
Individualised visualisation of the model using Shapley Additive Explanations. Predictions for two patients, showing correctly classified negative versus positive aggressiveness in patients with pancreatic neuroendocrine neoplasm, respectively. The colour gradient represents feature contributions, with yellow indicating a positive contribution and purple indicating a negative contribution.

SHAP force plots visualised individual predictions using the logistic regression model and accurately interpreted the predictions for two patients (**Figure 7**). The SHAP values are represented as forces that either increase or decrease the prediction. Each feature is shown by an arrow originating from the baseline value (0.406), which represents the average SHAP value across all predictions. The length of the arrow reflects the magnitude of the feature’s contribution (as a percentage), while the direction and colour (yellow for positive and purple for negative) indicate whether each feature increases or decreases the model’s prediction relative to the baseline.

## 4 Discussion

This study developed and evaluated an interpretable ML model integrating quantitative and qualitative CT imaging features to predict PNETsaggressiveness with high accuracy. Seven ML techniques were applied to a large patient cohort to minimise overfitting. Among these, logistic regression demonstrated the highest predictive performance, achieving AUCs of 0.952 and 0.972 in the training and test sets, respectively, demonstrating strong predictive efficacy. SHAP analysis enhanced interpretability, offering insights into key predictive factors.

Few studies have investigated PNETs aggressiveness. In one study, patients developed a deep learning-based nomogram using contrast-enhanced ultrasound (25), achieving AUCs of 0.97 and 0.85 in the training and validation sets, respectively, demonstrating strong discrimination, favourable calibration, and clinical utility. Mori et al. (26) predicted distant metastasis and microvascular invasion using radiomics features and clinical parameters, with AUCs of 0.67–0.85 and negative predictive values of 0.75–0.98. Shen et al. (27) identified tumour size, bile and pancreatic duct dilatation, lymphadenopathy, and enhancement patterns as key predictors, achieving AUCs of 0.89 and 0.86 in the training and test set. Despite these contributions, the lack of interpretability in previous models results in a “black-box” effect, which hinders clinical adoption (28).

To address this, our study employed SHAP analysis to enhance model interpretability while maintaining optimal predictive performance. SHAP quantified the contributions of four independent predictive features, determining their impact on tumour aggressiveness. Additionally, conventional CT imaging data were used, which are readily accessible without requiring complex software or invasive procedures. Compared to radiomics-based approaches, our model offers lower costs, reduced workload, superior predictive performance, and improved clinical applicability.Our findings highlight the potential of qualitative and quantitative CT features in PNETs risk stratification, although direct comparison with previous studies is not feasible. By providing both global and local interpretability, the model enhances transparency, allowing clinicians to better understand and trust its predictions.

This study identified four CT features as independent predictors of PNETs aggressiveness, ranked by SHAP analysis: tumour shape, arterial relative enhancement ratio, necrosis-cystic changes, and enhancement pattern. Irregular tumour margins were strongly associated with invasion and lymphatic spread, aligning with previous studies. Irregular margins are significantly correlated with tumour size, high histological grade, liver metastases, and lymph node metastasis (29). Zhu et al. (30) reported that tumours with irregular margins were 4.72 times more likely to develop malignant lymph nodes compared to those with smooth margins, further reinforcing the association between irregular borders and PNETsaggressiveness. Irregularly shaped tumours with incomplete capsules exhibit a higher likelihood of lymphatic or vascular invasion, which is strongly associated with poor prognosis, increased metastasis risk, and greater treatment resistance, significantly reducing overall survival (29).

Our findings indicate the critical role of necrotic changes in PNETs aggressiveness. Histopathologically, well-differentiated PNETs typically consist of small or medium-sized monomorphic cells arranged in trabeculae or islets, displaying a classic “salt-and-pepper” chromatin pattern. By contrast, poorly differentiated PNETs exhibit pleomorphic cells, highly atypical clusters, and extensive haemorrhagic necrosis (31, 32). Worhunsky et al. (33) similarly reported a relationship between hypodense foci and necrosis on histopathology. Some studies have also identified tumour size as a factor influencing PNETs aggressiveness (25, 34). In our study, although invasive PNETs were markedly larger, tumour size was not an independent risk factor, consistent with Chen et al. (35). Since most PNETs are asymptomatic and detected incidentally, symptom severity does not necessarily correlate with tumour size. Therefore, size alone is insufficient for assessing tumour aggressiveness.

The arterial relative enhancement ratio emerged as a critical predictor of tumour aggressiveness. The SHAP dependence plot demonstrated a strong inverse relationship: as the enhancement ratio increased, tumour invasiveness decreased. This may be attributed to PNETs being more susceptible to vascular invasion and microthrombosis, leading to reduced arterial supply and venous drainage (34). Differences in blood flow and microvascular density between tumours of varying invasiveness suggest angiogenesis levels are influenced by cellular differentiation and angiogenesis marker expression (36). High-grade PNETs frequently exhibit apoptotic bodies, map-like necrosis, and invasive growth patterns, resulting in low, heterogeneous enhancement (37). Our findings support the link between reduced microvascular density and arterial-phase enhancement, aligning with Yano et al. (38), which reported lower enhancement in metastatic tumours compared to non-metastatic ones.

Additionally, the arterial relative enhancement ratio provided a more objective and reliable prediction of aggressiveness than subjective radiologists’ assessments. As tumours progress, their imaging features become more complex, reducing the reliability of visual assessments. Thus, relying solely on radiologists’ expertise to predict aggressiveness has limitations. Further analysis revealed no significant differences in absolute enhancement, relative enhancement ratios, or portal phase enhancement across tumours of varying aggressiveness, reinforcing the greater predictive value of arterial phase enhancement. Collectively, these findings support the incorporation of CT features into a nomogram for accurate, preoperative PNETsrisk assessment.

After clinicians understood how various features influenced the logistic model and the potential pathophysiological mechanisms, they sought to apply the model to individual patient outcomes. To achieve this, we interpreted individual patient assessments locally by using the SHAP force plot. Compared to the nomogram method, which requires clinicians to calculate specific feature values to generate total points (39), the SHAP force plot is more time-efficient and user-friendly. Clinicians could directly compare the output SHAP value of a single patient with the base value, facilitating a clearer and more intuitive interpretation.

Our study has several limitations. First, Its retrospective, single-institution design may introduce selection bias and limit generalisability. Second, Given the rarity of PNETs, the cohort size—particularly the number of aggressive cases in the validation set—was relatively small, although measures such as LASSO feature selection and multivariate logistic regression were taken to minimise overfitting risks and ensure robust model performance. Third, we focuses on conventional CT features and did not incorporate radiomics analysis, which could potentially provide additional predictive information. Fourth, due to the extended recruitment period, various CT scanners were used, which may have affected image analysis. Fifth, while LASSO and multivariate regression helped control feature selection and combined effects, multiple comparison corrections (e.g., Bonferroni or FDR) were not applied, so a potential risk of Type I error remains. Despite these limitations, our findings demonstrate the potential of conventional CT-based features for predicting PNETs aggressiveness, providing a foundation for future multicenter studies that may integrate radiomics and further validate these predictive models.

## 5 Conclusion

In conclusion, we developed a reliable, interpretable ML model that accurately predicts PNETsinvasiveness, offering valuable insights into tumour biology. This model could aid preoperative decision-making and support personalised management strategies for patients with PNETs.

## Data Availability

The raw data supporting the conclusions of this article will be made available by the authors, without undue reservation.
